# Differences in Life History Traits in Rural vs. Urban Populations of a Specialist Ground Beetle, *Carabus convexus*

**DOI:** 10.3390/insects12060540

**Published:** 2021-06-10

**Authors:** Tibor Magura, Szabolcs Mizser, Roland Horváth, Dávid D. Nagy, Mária Tóth, Réka Csicsek, Gábor L. Lövei

**Affiliations:** 1 Department of Ecology, University of Debrecen, Egyetem sq. 1, H-4032 Debrecen, Hungary; mizserszabolcs@gmail.com (S.M.); horvath.roland@science.unideb.hu (R.H.); nagydavin@gmail.com (D.D.N.); toth.maria@science.unideb.hu (M.T.); csreka994@gmail.com (R.C.); 2 Department of Agroecology, Aarhus University Flakkebjerg Research Centre, DK-4200 Slagelse, Denmark; gabor.lovei@agro.au.dk

**Keywords:** environmental stress, carabid, body length, body mass, body condition, eggs, density-dependent fecundity

## Abstract

**Simple Summary:**

Urbanization is an important driver of global change, with negative consequences for biodiversity. Specialist species living in isolated urban forest fragments may be the most impacted by urbanization-driven environmental modifications. We compared various life history parameters of a forest specialist ground beetle in its original forest habitat and in urban forest fragments. Abundance was more than five times higher in the rural forest stands than in the urban forest fragments. We found no significant differences in body size or condition between the rural and urban individuals of either sex. Despite higher temperatures in urban habitats, the beginning of the reproductive period did not start earlier in the urban than the rural habitat. The number of ripe eggs was significantly higher in urban than rural females. The urban environmental conditions, however, seemed to cause high mortality of the immature stages, preventing the growth of urban populations.

**Abstract:**

Urbanization is increasing worldwide and causes substantial changes in environmental parameters, generating various kinds of stress on arthropods, with several harmful consequences. We examined a forest specialist ground beetle, *Carabus convexus,* in forested habitats to evaluate the changes in four important life history traits between rural and urban populations. Analyzing beetles from the overwintered cohort in their first breeding season, we found no significant differences in body length or body mass between the rural and urban individuals. Body condition, judged by fat reserve scores, was similarly poor in both habitats, indicating that beetles were not able to accumulate substantial fat reserves at either habitat. Females with ripe eggs in their ovaries were first captured at the same time in both areas. The number of ripe eggs, however, was significantly higher in females of the low-density urban population (6.13 eggs/female) than in those of the high-density rural population (4.14 eggs/female), indicating density-dependent fecundity. Altered environmental and habitat conditions by urbanization, however, seemed to cause high mortality during egg hatching and/or larval development, preventing the growth of the urban population to the level of rural one.

## 1. Introduction

Anthropogenic activities, including agriculture and urbanization, are important drivers of global change, with negative consequences for biodiversity [1]. Urbanization includes the spatial expansion of urban land use, and the growth of urban populations [2]. Already, 55% of the global human population lives in urban areas, and this is projected to increase in the following decades [3,4].

Urbanization is a human niche-creating activity, resulting in land take, resource concentration, and modulated environmental and ecological processes, from climatic regimes [5] to hydrological cycles [6] and biodiversity [7]. Many of the resulting effects are, however, detrimental to humans, directly or indirectly, including deposition of pollutants [8], reducing biodiversity [9], and/or various biological processes, like decomposition [10] or gene flow [11].

Urbanization-related processes cause modifications in habitat structure, composition, and environmental parameters but can also result in urban ecological novelties (UEN, [12]). The former may generate various kinds of stress on living organisms (on both vertebrates and invertebrates), with consequences on their activity pattern, spatial distribution, phenology, body condition, fecundity, behaviour, and biotic interactions [2,13,14]. UEN could provide novel resources for urban-dwelling organisms but can also lead them into an “ecological trap”, which is a preferred habitat that nevertheless results in a cost to fitness [15].

Urbanization is, without doubt, an important element of human-induced rapid environmental change [16,17]. However, cities can also play an important role in biodiversity conservation, because a multitude of species (e.g., 30% of birds and 5% of plants of global diversity), including both endemic and endangered ones, can still be found in urban areas [18,19].

Consequently, urban diversity matters, and in order to maximize the possible economic (e.g., ecosystem services), psychological, and environmental benefits that cities can provide, we have to understand how various urban dwellers fare in such environments, which conditions amount to hard environmental filters for them, and how to ease the negative impact these filters may cause. This task requires the examination of the effects of urbanization at various levels of biological organization, from genes to populations and communities [13]. Such studies have mostly been performed on vertebrates (mammals: [20,21], birds: [22,23]) and plants [24,25]. Not so numerous has been studies on terrestrial arthropods (but see [26]); nonetheless, urbanization is also seen as a global threat to insect diversity [27].

Of the terrestrial arthropods, ground beetles (Coleoptera: Carabidae) are a favorite group in urbanization studies [28,29,30] because of their diversity, abundance, and the availability of simple study methods [31]. Processes, environmental stress, and resource reorganization accompanying urbanization generate effects on ground beetles at manifold levels of biological organization [13]. Higher organizational levels may show intricate changes in patterns, but the interpretation of these patterns is challenging [32]. Change is the statistical product of the separate lower-level responses of a large number of individuals that cope as best as they can with the ever-changing conditions. Therefore, causative relationships are more appropriately examined with population-level studies. In population-level studies, life history and functional traits are useful to consider, because they may allow to arrive at acceptable generalizations [33].

Body size is possibly the most important trait, influencing many aspects of life history (development time, reproduction rate, dispersal power), and it also has a substantial impact on resource use, resource partitioning, and thus, on biotic interactions [34]. Trends in body size of ground beetles along urbanization gradients are contradictory. A decrease in body size from the rural area toward the urban one has been reported in some cases [35,36], while others have shown an opposite pattern: increasing body size with advancing urbanization [37,38]. Urbanization-induced environmental changes can also influence body condition, assuming better conditions in rural than urban habitats [39]. However, while several species indeed show better conditions in rural habitats than in urban ones, others show the opposite trend [39].

Habitat affinity has been claimed to determine species’ responses to urbanization-driven changes [28,40]. To date, however, there has been no study considering life history traits of habitat specialist ground beetle species in closely related rural and urban habitats. In the present paper, we aimed to fill this gap, by testing the following hypotheses on a forest specialist carabid, *Carabus convexus* Fabricius, 1775 (Coleoptera: Carabidae), in the city of Debrecen, eastern Hungary:

**Hypothesis 1 (H1).**  *Changes in environmental parameters and habitat characteristics accompanying urbanization may have significant effects on the diversity, quantity, and quality of food items [9]. We assumed that feeding conditions for carabid larvae would be inferior in urban vs. rural habitats. Given that larval conditions determine adult body size, we hypothesized that adults living in urbanized habitats would be smaller than those in non-urbanized ones.*

**Hypothesis 2 (H2).**  *The differences in food availability and quality could remain relevant for adults, and individuals living in more urbanized habitats may be in worse conditions, as found in the case of birds [41]. A suitable parameter to characterize body condition is the amount of fat reserves [42]. We hypothesized that urban-living individuals would have lower fat reserves than their rural conspecifics.*

**Hypothesis 3 (H3).**  *In connection to H2, we predicted that females in urbanized habitats cannot invest as much in reproduction as their rural conspecifics. Consequently, females should produce fewer eggs in urbanized habitats compared to rural ones.*

In the present study, we found no significant difference in body size or condition between the rural and urban individuals of either sex. However, the number of ripe eggs was significantly higher in urban than rural females.

## 2. Materials and Methods

### 2.1. Study Area and Sampling Design

Our study area lies on the eastern part of the Hungarian Plain, in and near the city of Debrecen (47°32′ N; 21°38′ E). From the extensive lowland forest near the city, we selected four rural forest stands. All selected forest stands (with sizes of 3.71 ha, 3.75 ha, 3.94 ha, and 3.04 ha, respectively) belong to a once-continuous old growth lowland forest (>100 years) dominated by English oak (*Quercus robur*), and recently, they have been embedded within an agricultural matrix with undisturbed or moderately disturbed habitats (e.g., meadows, grasslands, pastures, agricultural lands). Additionally, we selected four urban forest fragments (with sizes of 3.21 ha, 3.86 ha, 3.98 ha, and 3.33 ha, respectively) that were located inside the city. These are fragmented and isolated patches of the old growth lowland forest. Sites in the rural and the urban areas were at least 250 m from each other (mean distance between the urban sites: 702.2 m, distance between rural sites: 396.5 m). In the rural, continuous forest, there was no built-up area, while in the urban area, >60% of the surface within a 1000 m radius circle was built-up or was drastically different from the original forested habitat. Other, not quantified types of disturbances (presence of people, frequency, and intensity of habitat management/maintenance operations) also varied between areas. In the rural forest stands, there was no regular forestry intervention, and the presence of people was minimal, while in the urban forest fragments, the larger fallen branches and trunks were cut into smaller pieces and left on the ground, and the shrub layer was strongly thinned. Paths were asphalt- or gravel-covered, and human disturbance was considerable. The minimum distance between the rural and urban areas was 3 km [43,44].

Ground beetles were collected using 15 live pitfall traps per site, resulting in a total of 120 traps (2 areas × 4 sites × 15 traps). The location (coordinates) of the traps in sites were determined using a random number table, but ensuring that traps were installed at least 10 m apart from each other and at least 50 m from the nearest forest edge in order to avoid edge effects [45]. Traps were square plastic containers (170 mm long × 110 mm wide × 105 mm deep) containing shredded leaves to reduce predation on small, trapped arthropods by larger ones. Traps were covered with a 20 cm × 20 cm piece of fiberboard to protect them from litter and rain. Live trapping was conducted in the two main activity periods of ground beetles in the northern temperate region [31], between 6 April and 29 June (spring) and 31 August and 29 October 2020 (autumn). Traps were controlled twice per week. Trapped beetles were transported into the laboratory, identified to species level, and sexed.

### 2.2. Test Organism

*Carabus convexus* is a very widespread Eurasian species, with mainly nocturnal activity [46]. In Central Europe, this predator reproduces in early spring, and becomes active during the first half of April. In general, oviposition takes place from the middle of April onwards. Teneral individuals appear in late July and are present through to early August. Young adults go to overwintering in November. This wingless (brachypterous), moderately large-sized (14–23 mm) species has limited dispersal power [46]. In Hungary, *C. convexus* is a protected species, and in the studied region (Great Hungarian Plain), it is a forest specialist species [47]. In previous studies, *C. convexus* was categorized as a species very sensitive to urbanization [29,48]. In the studied location, its occurrence in urban forest fragments is sporadic and its abundance is also significantly lower than in rural forest stands [43]. The aforementioned characteristics (habitat specificity, large size, limited dispersal power) suggest that *C. convexus* may be a potential candidate as an indicator species of the effects of urbanization [13].

### 2.3. Evaluating and Measuring Traits

In the laboratory, individuals were immediately weighed twice by an analytical balance with precision of 0.1 mg (wet body mass). After weighing, beetles were stored in a freezer at −17 °C. Later, as a proxy for body length [42], elytral length (from the lower end of the scutellum to the apex of the elytra) of all individuals was measured three times with precision of 0.001 mm. Subsequently, mean body mass and mean body length were used as proxies for body size, although body mass and body length of ground beetles are correlated [49,50]. We also tested the relationship between the body mass and body length by a linear model. All beetles were aged based on mandible wear [51] and dissected to assess their body condition and to count the number of ripe eggs in the ovaries of females. The number of ripe eggs in the ovaries is a good proxy for fecundity and reproductive investment [31]. Body condition was characterized by the amount of fat content using a three-level scale (1–3), where 1 denoted no or very few, 2 moderate, and 3 large amounts of fat [52].

### 2.4. Statistical Analyses

The effect of urbanization (non-urbanized vs. urbanized) on the selected life history traits was tested using generalized linear mixed models (GLMMs) using the lme4 package [53]. The probability distribution that best fitted our response variable was checked using the car [54] and the MASS [55] packages. Based on these examinations, we modelled the response variables with count data (number of eggs, body condition) using a Poisson distribution with log-link function, while the other response variables (body mass, body length) were analyzed using normal error distribution with log-link function [56]. Fixed effects included urbanization level, sex of the tested individual (except for number of eggs), as well as their interaction. In the models, we also considered the nested design of our sampling (sampling sites were nested within the sampling areas). When GLMM revealed a significant difference between the means, the LSD test was performed for multiple comparison among means [56].

## 3. Results

We sampled 82 *C. convexus* individuals in the studied habitats. All individuals had sharp or very little worn mandibles, indicating that they were individuals from the overwintered cohort in their first breeding season. Sixty-nine beetles (35 females and 34 males) were caught at the rural sites, while 13 adults (8 females and 5 males) were caught in the urban sites. 

**Figure 1:**
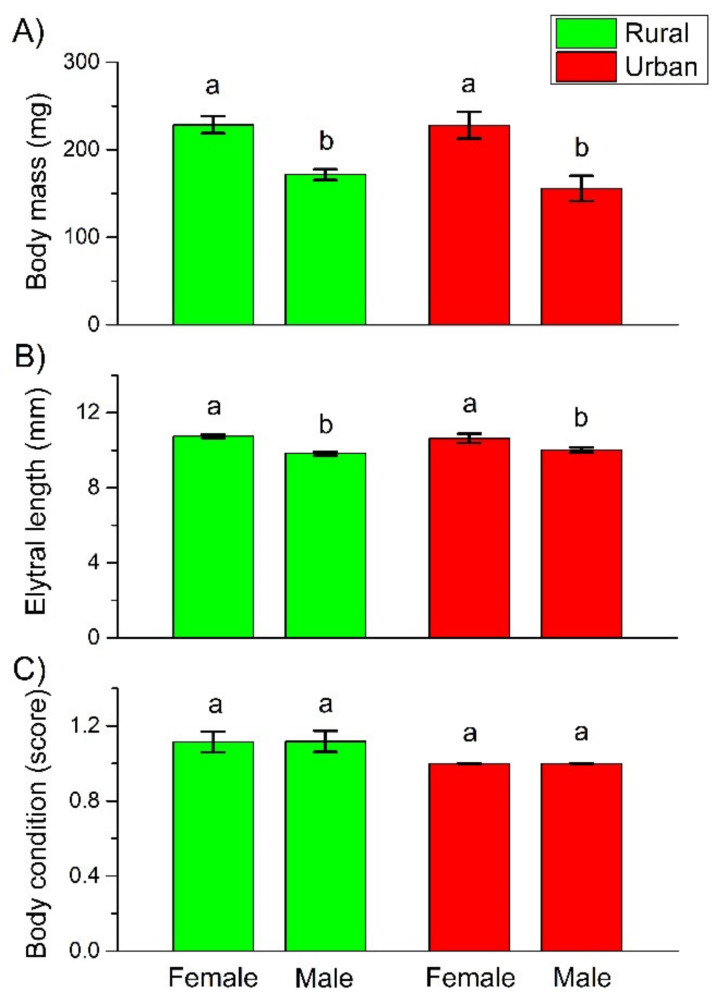
Mean (± SE) body mass (**A**), elytral length (**B**), and body condition, characterized by the amount of fat content using a three-level scale, where 1 denotes few, 2 moderate, and 3 large amount of fat content (**C**), of *C. convexus* individuals sampled in rural and urban habitats. Different letters indicate significant differences based on the LSD test (*p* < 0.05).

Both body mass and body length of the female beetles were significantly higher than that of the males. However, there were no significant differences, either between the individuals in habitats with different urbanization levels (rural vs. urban), or between individuals at the urbanization level × sex interaction (Table 1 and Figure 1A,B). Body mass and body length were significantly correlated (Figure S1). As neither the body mass nor the body length of rural females or males was significantly different from their urban counterparts, we did not include body mass or length as either a fixed (explanatory) or a random factor in the further GLMMs on life history traits. 

Body condition was bad in both the rural and urban habitats, regardless of the sex of the studied beetles. Furthermore, the interaction of urbanization level and sex had no effect on the body condition, either (Table 1 and Figure 1C). All these results indicated that *C. convexus* females and males were not able to accumulate substantial fat reserves in either the rural or urban forested habitats.

**Figure 2:**
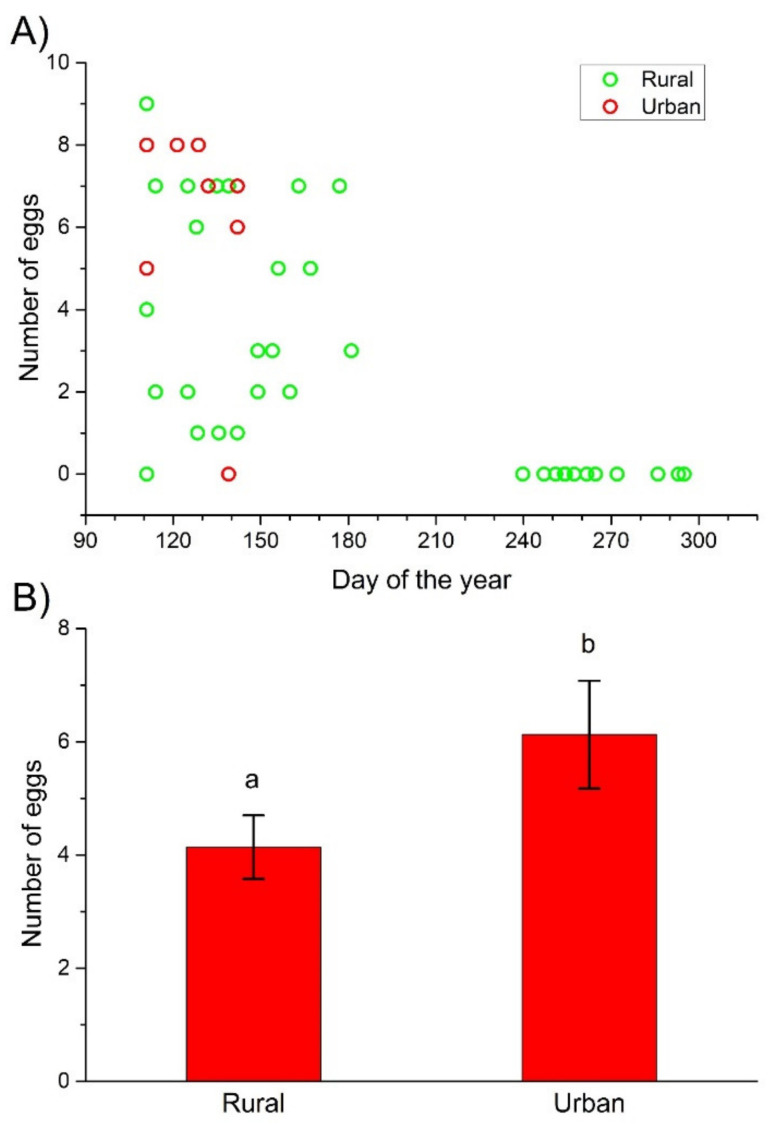
The number of ripe eggs in the ovaries of rural and urban *C. convexus* females during the days of the study year (**A**) and the mean number (±SE) of ripe eggs in the ovaries of females before oviposition trapped between April and June 2020 in the rural and urban habitats (**B**). Different letters indicate significant differences based on the LSD test (*p* < 0.05).

Based on the trapping data (trapping begun on the 97th day of 2020), the appearance of females with ripe eggs in ovaries was not different between the rural and urban habitats, as females with ripe eggs were first captured on 20 April (the 111th day of the year) in both areas (Figure 2A). The number of ripe eggs in the ovaries, however, was significantly different between the rural and urban females, regardless of whether all sampled females or only females before oviposition (females trapped from April to June) were considered (Table 1). Urban females had significantly more eggs in their ovaries than their rural counterparts (Figure 2B).

## 4. Discussion

### 4.1. Beetle Abundance

We trapped more than five times as many *C. convexus* individuals in the rural forest stands as in the urban forest fragments. This difference proves the significant sensitivity of this species to changes in environmental parameters and habitat characteristics accompanied by urbanization [29,47]. In previous studies at the same urban area, no [44] or only a few [43] individuals were sampled using pitfall traps with preservatives. These findings indicate the sporadic occurrence of this species in the studied urban forest patches. In the present study using live pitfall traps, however, many more individuals were sampled in the urban habitats compared to earlier studies using traps with preservatives. Although live-trapped beetles can attract their peers, increasing the number of individuals caught, a comparative study found no significant difference in the number of individuals trapped between traps with a killing agent and live pitfall traps [57]. Therefore, the higher number of trapped beetles than before may indicate annual fluctuations in population size of *C. convexus* in the studied urban habitats. However, due to the large difference in the number of sampled beetles in the two habitats, our results should be interpreted with caution.

### 4.2. Body Mass, Length, and Condition

Contrary to our hypothesis, the body size (expressed by either body mass or body length) of *C. convexus* females and males were not significantly different between the rural and urban habitats. Environmental stress can decrease body size, so it should decrease from less disturbed to more disturbed habitats [58,59], i.e., in the present context, from rural to urban areas. Furthermore, physiological constraints may also shape body size pattern along the urbanization gradient. Recently, it was predicted that the urban heat island effect drives shifts towards smaller body sizes in urban communities of ectotherm species, in line with Atkinson’s temperature–size rule [60,61]. This physiological constraint proved to be strong in habitat specialist species [30]. Moreover, increasing stress/disturbance was predicted to be detrimental to large-sized species because of their low reproductive output, smaller population size, larger home range, lower dispersal power, and longer life cycle [31,62]. *C. convexus* was not a dominant member of the studied assemblages; therefore, Gray’s stress hypothesis, predicting reduced body size of the dominant species of the assemblages in disturbed habitats [59], may not apply to it. However, this species is a large-sized and strictly forest specialist species in the studied region; thus, the physiological constraint should affect its body size along the urbanization gradient. Previous results on changes in body size of ground beetle species along urbanization gradients are also inconsistent, even for studies performed at the same localities. In and around Hamburg city (Germany), of the four studied habitat generalist species, only the adult size of *Carabus nemoralis* O.F. Müller, 1764, a large-sized subdominant species in the given assemblage, decreased significantly from the rural area towards the city center [35]. In Birmingham city (UK), on the contrary, adult body size of two dominant and forest associated species, *Pterostichus madidus* (Fabricius, 1775) and *Abax parallelepipedus* (Piller & Mitterpacher, 1783), increased significantly with increasing urban cover [38]. Based on the above, it seems that the strength and intensity of environmental stress, the size, or habitat specificity of the studied species cannot be considered as strong, unidirectional factors influencing beetle body sizes in urban habitats. Other factors, such as the sensitivity of immature life stages (eggs and larvae), microhabitat requirements, and the feeding specificity of larvae and adults, could be more important factors [13,31].

Contrary to our hypothesis, urban individuals were not in worse condition (did not have lesser fat content) than their rural conspecific. Body condition (characterized by amount of visible fat) of females and males was similarly bad in both habitats, suggesting that individuals could not accumulate large fat reserves. Ground beetles can consume close to their own body mass of food daily. This food, especially before reproduction and hibernation, is used to build fat reserves [31]. Under field conditions, however, ground beetles are frequently captured with empty guts. Therefore, food limitation under field conditions exists for larvae [63] as well as adults [64,65]. Furthermore, food shortage can be aggravated by inter-and intraspecific competition between ground beetles [64,66], as well as by intraguild competition between ground beetles and other ground-dwelling generalist predators, like spiders and ants [31]. Our results showed that ground beetles could be under constant food shortage, regardless of the degree of urbanization.

### 4.3. Fecundity

Feeding conditions during larval development basically determine adult size, which is a key determinant of potential fecundity. Realized fecundity, however, also depends on the feeding conditions of the adult [31]. Indeed, fecundity of adults markedly increases with increased food supply and feeding rate [52,67]. Not only the quantity, but also the quality of food influences reproduction in ground beetles. A diverse diet, shifting between carbohydrate-rich and protein-rich food, increases the egg number of ground beetles under laboratory conditions [68]. Females feeding under field conditions, however, realized only about half of their possible maximum egg production, indicating food limitation on reproduction [31]. We predicted that females in urbanized habitats were in a worse condition and could not invest as much into reproduction as their rural counterparts, thus producing fewer eggs. The findings did not support this prediction: the egg numbers were significantly higher in urban than in rural females. This pattern is consistent with the density-dependent fecundity hypothesis driven by competition for food, predicting that egg production is inversely correlated with population size [67], as the probable population size of *C. convexus* (expressed as the total catch) in urban forest fragments was considerably lower than that in rural forest stands. However, the adult population in the urban fragment was still scarce, either because the environmental and habitat conditions created by urbanization caused high mortality during immature development, or the species only recently has colonized the urban study area. Moreover, habitat fragments in urbanized areas frequently become isolated, making the between-patch dispersal of individuals difficult or even impossible [11]. Urban habitats, however, may have more prey and fewer predators [13,14], having beneficial effects on body size, body mass, and condition. Under reduced predation pressure and competition, urban beetles could be larger and have greater fat reserves, but these were not observed. Alternatively, the urban females were allocating more resources to egg production, leaving their body condition in the same bad state as the rural females, but with more eggs.

### 4.4. Conserving Ground Beetles in Urban Areas

Our results show that despite the higher fecundity of urban females, the studied ground beetle species had significantly lower abundance in urban forest fragments than in rural forest stands. Altered habitat characteristics, environmental parameters, fragmentation, and isolation effects by urbanization could be the main reasons for this low abundance [7,13,27]. Therefore, intense urban habitat management (frequent mowing, strong thinning and pruning, and removal of decaying wood) should be eased or abandoned. Positive effects of adopting soft management practices on ground beetles are well documented [69]. Mitigating the effects of fragmentation and isolation, the maintenance and/or restoration of source habitats, and the preservation or creation of corridors or stepping stones are key issues for promoting species dispersal between urban fragments and species survival in these patches [7,13,70]. Thus, to conserve and restore biodiversity in urbanized areas, a holistic, multi-scale management and planning scheme is urgently needed [7,71].

## 5. Conclusions

Comparing various life history traits of a forest specialist ground beetle, *C. convexus*, in rural forests and urban forest fragments, we showed that the species was more abundant in rural than urban habitats. Body size or condition were not significantly different between the rural and urban individuals of either sex, while the number of ripe eggs was significantly higher in urban than rural females. Our results suggest that urbanization-driven environmental changes may cause high mortality during egg hatch or larval development, preventing the increase of population size in urban forest fragments. Therefore, to preserve a self-sustaining population of the studied large sized, forest specialist species with limited dispersal power, adverse environmental changes by urbanization should be prevented or mitigated. To realize these goals, multi-scale greenspace planning and management schemes are needed [7,13].

## Figures and Tables

**Table 1:** Summary of GLMM results and post hoc tests on life-history traits of *Carabus convexus* in differently urbanized (non-urbanized vs. urbanized) forested habitats (*p*-values in bold denote significant effects).

Response Variable	Fixed Effect	Estimate ± SE	χ^2^	df	*p*
Body mass	Urbanization level	−0.0555 ± 0.1432	0.1499	1	0.6987
	Sex	0.2614 ± 0.0421	38.4839	1	**<0.0001**
	Urbanization level × Sex	0.0843 ± 0.1080	0.6081	1	0.4355
Body length	Urbanization level	0.0164 ± 0.0328	0.2498	1	0.6172
	Sex	0.0862 ± 0.0132	42.6806	1	**<0.0001**
	Urbanization level × Sex	−0.0267 ± 0.0336	0.6303	1	0.4272
Body condition	Urbanization level	−0.1112 ± 0.4757	0.0547	1	0.8151
	Sex	−0.0030 ± 0.2279	0.0002	1	0.9895
	Urbanization level × Sex	0.0030 ± 0.6140	0.0000	1	0.9961
Number of eggs					
All females	Urbanization level	0.9673 ± 0.4099	5.5702	1	**0.0183**
Females before oviposition	Urbanization level	0.3926 ± 0.1772	4.9082	1	**0.0267**

## Data Availability

The data presented in this study are available on request from the corresponding author.

## Supplementary Materials

The following are available online at https://www.mdpi.com/article/10.3390/insects12060540/s1, Figure S1: Relationship between the elytral length (as a proxy for body length) and the body mass of *C. convexus* individuals sampled in rural and urban habitats. The slope of the fitted line is significantly different from zero (adjusted R^2^ = 0.5912; F_1,80_ = 118.1375, *p* < 0.001). Equation of the fitted line: Body mass=−0.3994+0.0582×Elytral length.
