# Supplementary material for: Differences in Life History Traits in Rural vs. Urban Populations of a Specialist Ground Beetle, Carabus convexus
Source: Insects. 2021 Jun 10;12(6):540. doi: 10.3390/insects12060540 (PMC8230416; doi:10.3390/insects12060540)
Supplement: Supplementary file 1 [file insects-12-00540-s001.zip › insects-1236091-supplementary.pdf]

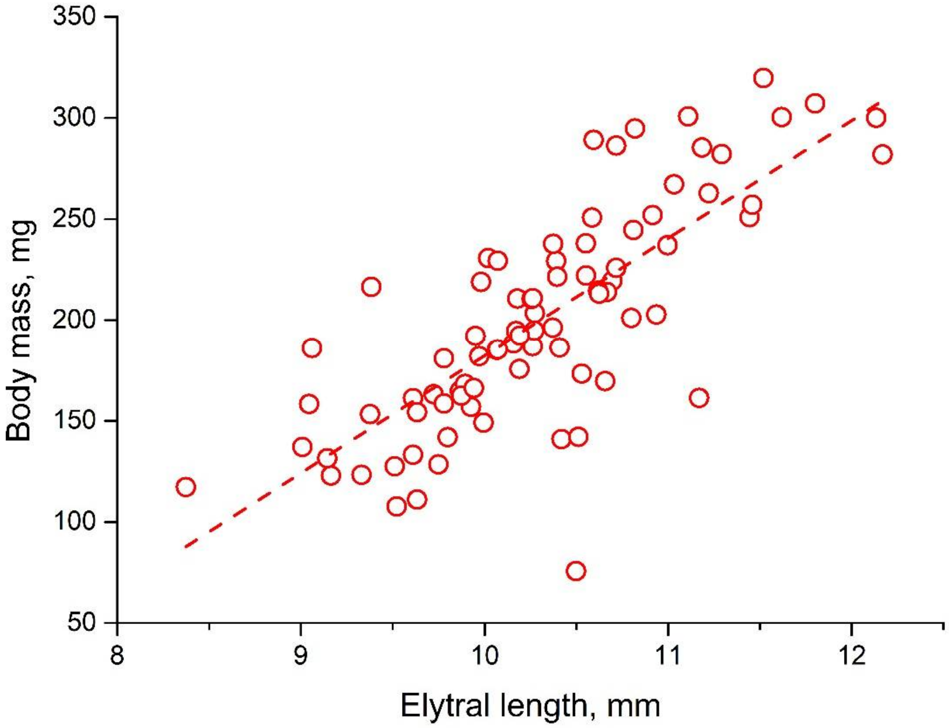

Figure S1. Relationship between the elytral length (as a proxy for body length) and the body mass of *C. convexus* individuals sampled in rural and urban habitats. The slope of the fitted line is significantly different from zero (adjusted R^2 = 0.5912; $F_{1,80}$ = 118.1375, p < 0.001). Equation of the fitted line: $Body\ mass = -0.3994 + 0.0582 \times Elytral\ length$
